# Investigation of *Dracocephalum* extract based on bulk and nanometer size as green corrosion inhibitor for mild steel in different corrosive media

**DOI:** 10.1038/s41598-023-27891-y

**Published:** 2023-01-17

**Authors:** Zahra Golshani, Faezeh Arjmand, Mahnaz Amiri, Seyed Mohammad Ali Hosseini, S. Jamiladin Fatemi

**Affiliations:** 1 Department of Chemistry, Shahid Bahonar University of Kerman, P.O. Box 76169-14111, Kerman, Iran; 2 Neuroscience Research Center, Institute of Neuropharmacology, Kerman University of Medical Science, Kerman, Iran; 3 Student Research Committee, Faculty of Allied Medicine, Kerman University of Medical Sciences, Kerman, Iran

**Keywords:** Nanoscience and technology, Chemistry, Electrochemistry, Green chemistry, Physical chemistry

## Abstract

In recent years, green corrosion inhibitors derived from natural plant resources have garnered much interest. In the present work, at first, we investigated the corrosion behavior of mild steel (st-37) in the presence, and absence of *Dracocephalum* extract based on bulk size as a corrosion inhibitor in two widely used acidic environments (0.5 M H_2_SO_4_, and 1.0 M HCl), at room temperature. Then, we used *Dracocephalum* extract based on nanometer size to reduce the optimal concentration of inhibitor, increase the corrosion resistant, and efficiency. *Dracocephalum* extract does not contain heavy metals or other toxic compounds, and also good characteristics such as low cost, eco-friendly, and widespread availability, make it suitable nature candidate as an environmentally safe green inhibitor. The anticorrosive behavior was assessed using electrochemical impedance spectroscopy (EIS), and potentiodynamic polarization (PP). In all of the studies, the inhibitory efficiency (*IE*%) increased as the extract dose was increased. But by using nano extract, in addition to maintaining high efficiency, the amount of inhibitor was reduced significantly. The highest *IE*% is 94% at the best dose of nano extract (75 ppm), but the highest *IE*% is 89% at the best dose of the bulk extract (200 ppm) in H_2_SO_4_ solution. Also, for the HCl solution, the highest *IE*% is 88% at the best dose of nano extract (100 ppm), but the highest *IE*% is 90% at the best dose of the bulk extract (400 ppm), by polarization method. The PP results suggest that this compound has an effect on both anodic, and cathodic processes, and that it adsorbs on mild steel surface according to the Langmuir adsorption isotherm. Optical microscopy, scanning electron microscopy (SEM) analysis, and a solid UV–Visible reflection spectrum were used to investigate the alloys' surface morphology.

## Introduction

Corrosion has claimed lives, and riches in practically every technical sector in the past^1^. Corrosion is defined as the deterioration of metals, and alloys as a result of chemical, and physical interactions with their surroundings. The anodic, and cathodic reactions are the chemical processes that create this behavior^2^. Not only that, but the expense of reviving corrosion-damaged manufacturing equipment contributed significantly to a country's gross domestic product. As a result, all hands must be on the desk to oppose this dangerous deed by doing a periodic study into its final resolution^1^.

Because of their great mechanical, and electrical qualities, metals are frequently utilized in human activities^3^. Mild steel is the most often used metal in major industrial businesses due to its cost-effectiveness, and excellent outstanding mechanical properties. However, because of its low corrosion resistance, particularly in acidic, and alkaline settings, its application has been restricted^4^. The utilization of acid solution in industrial applications has been chiefly used to study the occurrence of mild steel corrosion inhibition mechanisms in acidic environments. The refining process of crude oil, for example, results in various corrosive conditions. In most situations, refinery corrosion is caused by powerful acids attacking the equipment's surface^5^.

To avoid the corrosion of metals, many methods have been designed after analyzing the different forms of corrosion^2^. These methods include: inhibitors, electrical protection, surface coating, equipment design, and material selection^6^. Inhibitors are chemicals that, when applied in tiny amounts to corrosive conditions, inhibit electrochemical corrosion processes on metal surfaces^1,7^.

The use of corrosion inhibitors is a cost-effective way to reduce corrosion rate, shield metal surfaces against corrosion, and ultimately protect industrial equipment in harsh environments^8^. The inhibitors work at the interface between the corrosive aqueous solution, and the metal, influencing the electrochemical process procedures by adsorption on the metal's surface^9^. Polar functional groups^10^, which help to reduce the sensitivity of a metal surface to corrosion, are centers of reactivity that guarantee the stability of this adsorption process^11,12^.

Corrosion inhibitors have been extensively worked out in numerous industries to reduce the rate of dissolution of metal goods in contact with a damaging environment. The ability of corrosion inhibitors to adsorb on metal surfaces was linked to their high efficiency^13^.

Corrosion inhibitors' biodegradability, accumulation, and toxicity have all been questioned recently. Researchers' safety, environmental pollution, and economics are all significant concerns as researchers seek safe, non-polluting, and cost-effective inhibitors^14^.

Therefore, when selecting an inhibitor, several variables must be addressed, including cost, quantity, ease of availability, and, most importantly, safety to the ecosystem, and its species^15^.

In the recent decade, green chemistry has been attracting great interest in many contexts by commercial products, chemical technologies, and designing chemicals to reduce wastes, and avoiding toxins^16^. Green inhibitors are getting much attention in the corrosion field thanks to their renewability, ecologically acceptability, biodegradability, and safety^17^. These include, for example, polyphenols^18^, alkaloids^15^, amino acids^19^, and often extracts of plants^20^. As a result, scientists have been looking for green corrosion inhibitors that can retain high inhibitory efficiency while lowering toxicity in recent years^21^. Organic extracts having functional groups, including sulfur, nitrogen, and oxygen atoms in a conjugate system, are effective inhibitors^22^. Organic green corrosion inhibitors limit corrosion by eliminating water molecules from the surface of the metal/solution contact, resulting in the creation of a compact barrier layer^23^.

Nanostructured materials have been studied considerable because of their broad range of prominent applications because nanostructures exhibit novel size-dependent properties, such as magnetic and mechanical and chemical properties, that extensively differ from their bulk materials, that exhibit great potential in the novel fields^24^.

Several authors have reported using natural materials as corrosion inhibitors, such as extracted compounds from seeds or leaves. Gunasekaran et al.^25^ investigated the corrosion prevention of steel by the environmentally beneficial *Zenthoxylum alatum* plant extract in phosphoric acid. Corrosion inhibitors such as leaf extracts, and essential oils are commonly employed^26^. Corrosion inhibition of leaves extracts, and essential oils such as *Acacia Arabica*^27^, *Annona squamosa*^28^, *Rosmarinous officinalis*^29^, *Aloysia citrodora*^30^, and *Lawsonia*^31^, which were employed for steel in acid medium, was studied.

*Dracocephalum* is a genus of flowering plants in the *Lamiaceae* family with around 60^32^ to 70 species^33^ endemic to temperate parts of the Northern Hemisphere. These flowers, commonly known as dragonhead, are herbaceous perennials or subshrubs that grow to a height of 15 to 90 cm. This plant is widely utilized in contemporary medicine to treat a variety of viral disorders as well as to inhibit tumor progression over the world^34^. *Dracocephalum* has several biological, and pharmacological activities, including antibacterial^35^, antifungal^36^, and anti-inflammatory^37^.

*Dracocephalum* extract is a strong contender for use as an ecologically safe green inhibitor since it doesn't include heavy metals or other harmful substances. It also has favorable qualities including affordability, environmental friendliness, and wide availability. So, to overcome the disadvantages of widely used organic corrosion inhibitors, which are expensive and toxic to the environment, and in continuation of our previous works on the development of green corrosion inhibitors^3^, we report herein the inhibiting effect of *Dracocephalum* extract in bulk, and nanometer size on the corrosion of mild steel (st-37) in acidic media employing electrochemical impedance spectroscopy (EIS), and potentiodynamic polarization (PP) methods. In each study, the extract dosage was raised as the inhibitory effectiveness rose. However, employing nano extract significantly decreased the quantity of inhibitor while still retaining high efficiency. The experimental data obtained by optical microscopy, scanning electron microscopy, and UV–visible spectroscopy to confirm or reject the potential of this herbal extract as a novel green inhibitor. This article gives a vivid account of *Dracocephalum* extract as a natural product which is used as a corrosion inhibitor for mild steel alloy in aggressive media, with suitable efficiency, and the minimum concentration of inhibitor based on nanometer size.

## Experimental details

### Materials

Materials were commercially available and employed without further purification and prepared from Arshanzist Youtab Company. For the preparation of the electrolytes, and *Dracocephalum* extract, the following materials, and reagents were used: sulfuric acid (MW 98.08 g/mol, 96%), hydrochloric acid (MW 36.46 g/mol, 37%), ethyl alcohol (MW 46.07 g/mol, 99.5%), methanol (MW 32.04 g/mol, 99.8%), and distilled water (MW 18.02 g/mol).

### Preparation of the st-37 electrodes for electrochemical test

The samples for the corrosion testing were made of mild steel. Table 1 shows the chemical composition of the alloy.

**Table 1:** Chemical composition of mild steel (wt%).

Element	C	Mn	P	Si	Cr	Al	Cu	Fe
Wt%	0 0.076	0.192	0.012	0.026	0.050	0.023	0.123	Balance

Specimens with a surface area of 1 cm^2^ were used for all electrochemical experiments. The exposed side of the steel sheets was polished to a mirror shine with several grades of emery papers (100, 400, 1000, and 2500). Distilled water was used to clean the substrates, which were then degreased with ethyl alcohol and dried at room temperature.

### Preparation of *Dracocephalum* extract

The healthy leaves of *Dracocephalum* were purchased from the local markets in Iran, which are completely designated for commercial usage. To eliminate dust, the gathered leaves were gently washed. The leaves were dried in the shade at room temperature. At ambient temperature and in the dark, 100 g of dried *Dracocephalum* leaves were soaked in methanol for 72 h. The surplus solvent was evaporated under reduced pressure in a rotary evaporator at 40 °C after filtering the solution. The recovered residue had a consistent weight of 2.0 g.

It is noteworthy that, alcohol-based herbal preparations are those that use some form of alcohol as the solvent. Herbal tinctures, and herbal liniments are both considered alcohol-based preparations even though two different types of alcohol are used (ethyl alcohol and isopropyl alcohol, respectively). Alcohol preparations have a long shelf life as alcohol slows the decomposition of materials and bacterial growth, thus, increasing herbal preparation shelf life^38^.

### Declaration for the usage of plant materials

We declare that in this research, we did not use or not going to use any plants (either cultivated or wild) irrespective of any location. Experimental research and field study in this study has complied with the IUCN Policy Statement on Research Involving Species at Risk of Extinction. The use of plants in the present study complies with international, national and/or institutional guidelines.

### Preparation of Nanosized *Dracocephalum* extract

To obtain herbal nanostructures, the following method used. A specific value of pure *Dracocephalum* extract dissolved in 100 mL of ethanol in a beaker to have a solution. The solution stirred at room temperature via vigorous stirring for 30 min at 800 rpm, then the product filtered using filter papers (Whatman, 40 Ashless, Germany) to remove probable impurities. The filtered solution added at a 1:10 ratio to distilled water to isolate pure herbal particles. The suspensions placed in an ultrasonic bath for 20–30 min, and afterwards, to produce lower-sized nanostructures, ultra-prob sonication for 20 periods of 10 s (Hielscher, UP100H, Germany) used as well. Afterward, nanoparticles acquired in the colloid state. In this colloid, nanoparticles observed using dynamic light scattering (DLS) techniques.

### Preparation of solutions

The corrosive media were 0.5 M H_2_SO_4_, and 1.0 M HCl, made by diluting analytical grade Merck H_2_SO_4_, and HCl with double distilled water, respectively. Before to each experiment, the test solutions were made fresh by mixing the extract with the corrosive solution. Experiments were conducted twice to verify repeatability. The extract concentrations were 50, 100, 150, 200, and 250 ppm for 0.5 M H_2_SO_4_, and 100, 200, 300, 400, and 500 ppm for 1.0 M HCl based on bulk size, and 25, 50, 75, and 100 ppm for 0.5 M H_2_SO_4_, and 50, 75, 100, and 125 ppm for 1.0 M HCl based on nanosized extract.

Noticeably, pentacyclic triterpenoids are one of the main functional components in *Dracocephalum* extract. Pentacyclic triterpenoids are practically insoluble in water and low concentration ethanol, but they are soluble in chloroform, HCl, and acidic media^39^.

### Characterization

To investigate the size distribution or average sizes of the plant extract, dynamic light scattering (DLS) was employed. DLS data obtained using a Nano-ZS90 (Malvern) apparatus (Malvern Instruments, Malvern, UK). Electrochemical research such as electrochemical impedance spectroscopy, and potentiodynamic polarization were done using the AutoLab device (302 N potentiostat, Netherlands). Scanning electron microscopy (SEM FEI Quanta 200, accelerating voltage 20.0 kV), and optical microscopy (Leica zoom 2000 model) were used to investigate the surface morphology of mild steel submerged in sulfuric acid, and hydrochloric acid without and with the optimal concentration of *Dracocephalum* extract. The measurements of UV–Visible reflection spectra of surface species on the mild steel were performed by using UV–Vis spectrophotometer A SPECORD 210 (Analytik Jena, Germany) in the stainless tank (*π* × 1^2^ × 1.5 cm) to avoid interference from ambient light. This spectrophotometer is controlled with the Spectra Manager software. For the last two tests, working electrodes were mechanically polished, and immersed in 0.5 M H_2_SO_4_, and 1.0 M HCl solutions in the absence, and presence of an inhibitor for about 24 h at room temperature and then removed and dried.

### Statistical analysis

After exploring the normal distribution using the Kolmogorov-smearnov test, the data were subjected to One Way ANOVA, and Tukey Post Hoc tests (S = 0.05).

### Stability study

The synthesized nanoparticles were stored at 4 °C, room temperature (24 °C), and physiologic temperature (37 °C) for 3 weeks in the glass vials. After a duration of storage, the distribution of nanoparticles size considered to detect the variations in the formulation with respect to time.

## Procedures

### Electrochemical measurements

EIS is a vital way of monitoring in situ electrochemical changes with critical knowledge of physical processes occurring at the metal/electrolyte interface^40^, so impedance diagrams may provide information on mechanistic, surface characteristics, and electrode kinetics^41^. In most applications, the basic lab setup comprises employing three electrodes in the electrochemical cell for the measurement: working, counter, and reference electrodes submerged in a specified volume and the concentration test solution. So in this work, a three-electrode cell containing Pt electrode, Ag/AgCl electrode, and st-37 specimen as a counter, a reference, and a working electrode, respectively, have been used. First, the open circuit potential (OCP) was recorded for 30 min, and then the EIS data were obtained. The experiment is carried out using a modest potential of 10 mV of AC voltage and frequencies ranging from 100 kHz to 100 mHz. The inhibition efficiency (*IE*_*I*_) of a corrosion inhibitor was estimated using the following equation utilizing electrochemical data collected from the workstation^42^:

1 $$IE_I\% =\left(\frac{Rct-R{^{\prime}}ct}{{R}_{ct}}\right)\times 100$$

where *R*_*ct*_, and *R′*_*ct*_ are the polarization resistance of the sample in the presence, and absence of the corrosion inhibitor, respectively.

Potentiodynamic polarization is another electrochemical-based method for determining the corrosion mechanism protection, corrosion rate, and effectiveness of green corrosion inhibitors. The experiment is carried out in a three-electrode electrochemical cell, the same as EIS. The polarization scan rate was set at 1 mV/s to plot the Tafel polarization curves. The electrode potential was changed automatically from − 800 mV to − 100 mV vs. *E*_*corr*_ at 25 ± 1 °C to create these graphs. After EIS, a potentiodynamic test was used to determine the polarization curve. The corrosion inhibitor's inhibition efficiency (*IE*_*P*_) is calculated using the following equation^43^:

2 $$IE_P\%=\frac{{i}_{corr}-{i}_{corr}^{^{\prime}}}{{i}_{corr}}\times 100$$

where, *i*, and *i′* are the current densities of the solution in the absence, and presence of the inhibitor, respectively.

Also, using NOVA 1–10 software, the suitable equivalent circuit, the corresponding EIS, and potentiodynamic polarization parameters can be prepared.

To check the reproducibility of the results, at least two experiments were performed at each concentration for EIS, and potentiodynamic polarization curve. The standard deviations (S.D.) were obtained and, S.D. values were small, suggesting that the electrochemical measurements had good reproducibility. In this work, S.D. is smaller than 0.5 for all electrochemical experiments, so these data were omitted in the following sections.

## Results and discussion

### Characterization of the nanosized plant extract

**Figure 1:**
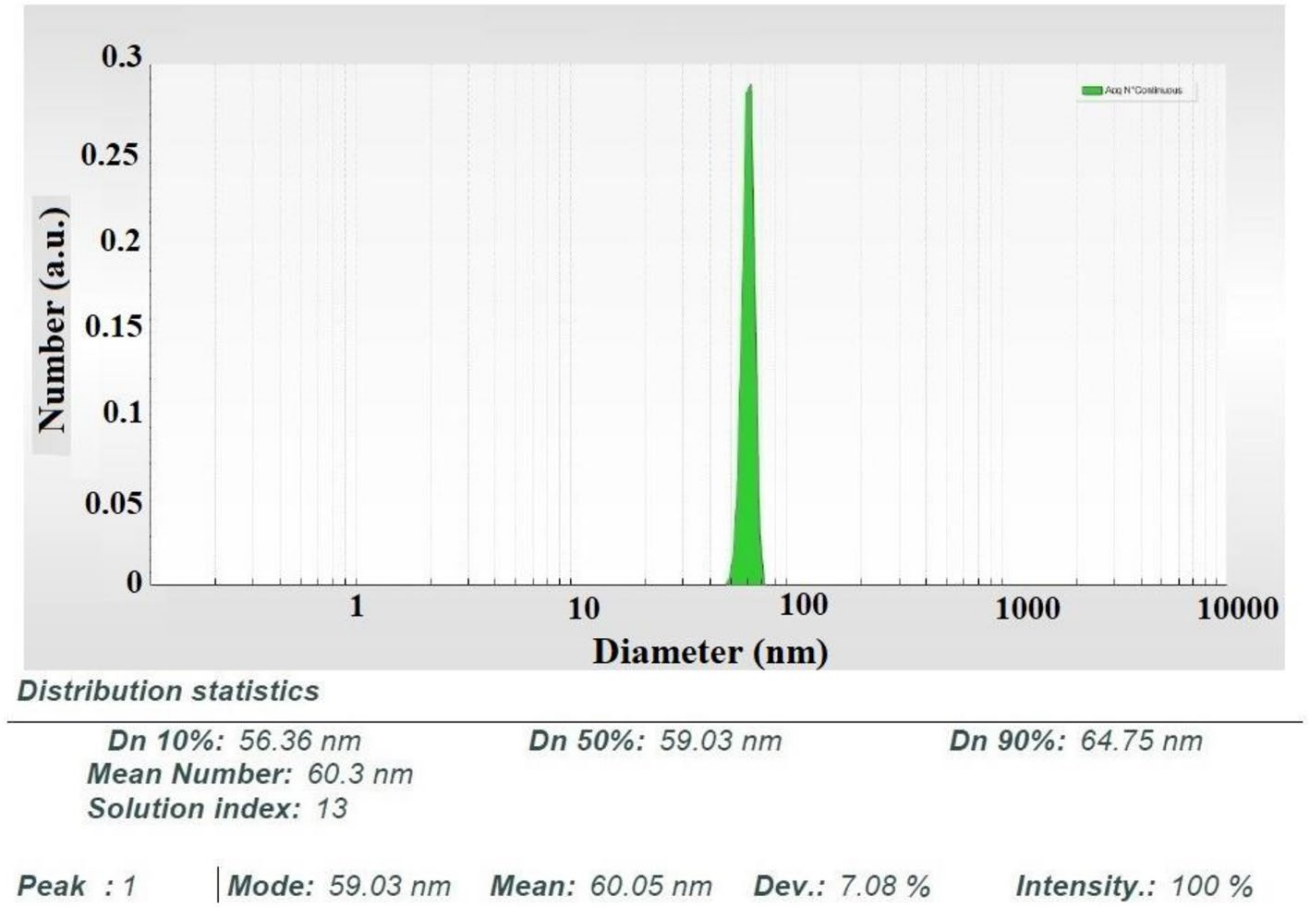
The mean size of produced nanoparticles recorded by the nanosizer equipment (DLS technique).

Dynamic light scattering is used for measuring average particle diameter, and particle diameter distribution of nanosized particles dispersed in the liquid. Extract Biomolecules like proteins, enzymes, terpenoids, and flavonoids cofactors play both capping, and reducing role. Furthermore, due to strong binding ability with amino acid residues (carbonyl group), agglomeration behavior was prevented, and stability of medium was provided. For a better understanding of the real size of nanoparticles, the nanosizer technique used for calculating particle size, and stated by SBL (Statistical Bin Limits) analysis. For this propose, reduction of agglomeration fault to state actual particle size was done by omitting hydrodynamic radius. Figure 1 reported the histogram of the SBL nanosizer of NPs, which showed the mean diameter of the size of the particle is ~ 64.75 nm for nanostructures. Reported results demonstrated narrow size distribution, and homogenous dispersity of NPs.

### Corrosion inhibition study

**Figure 2:**
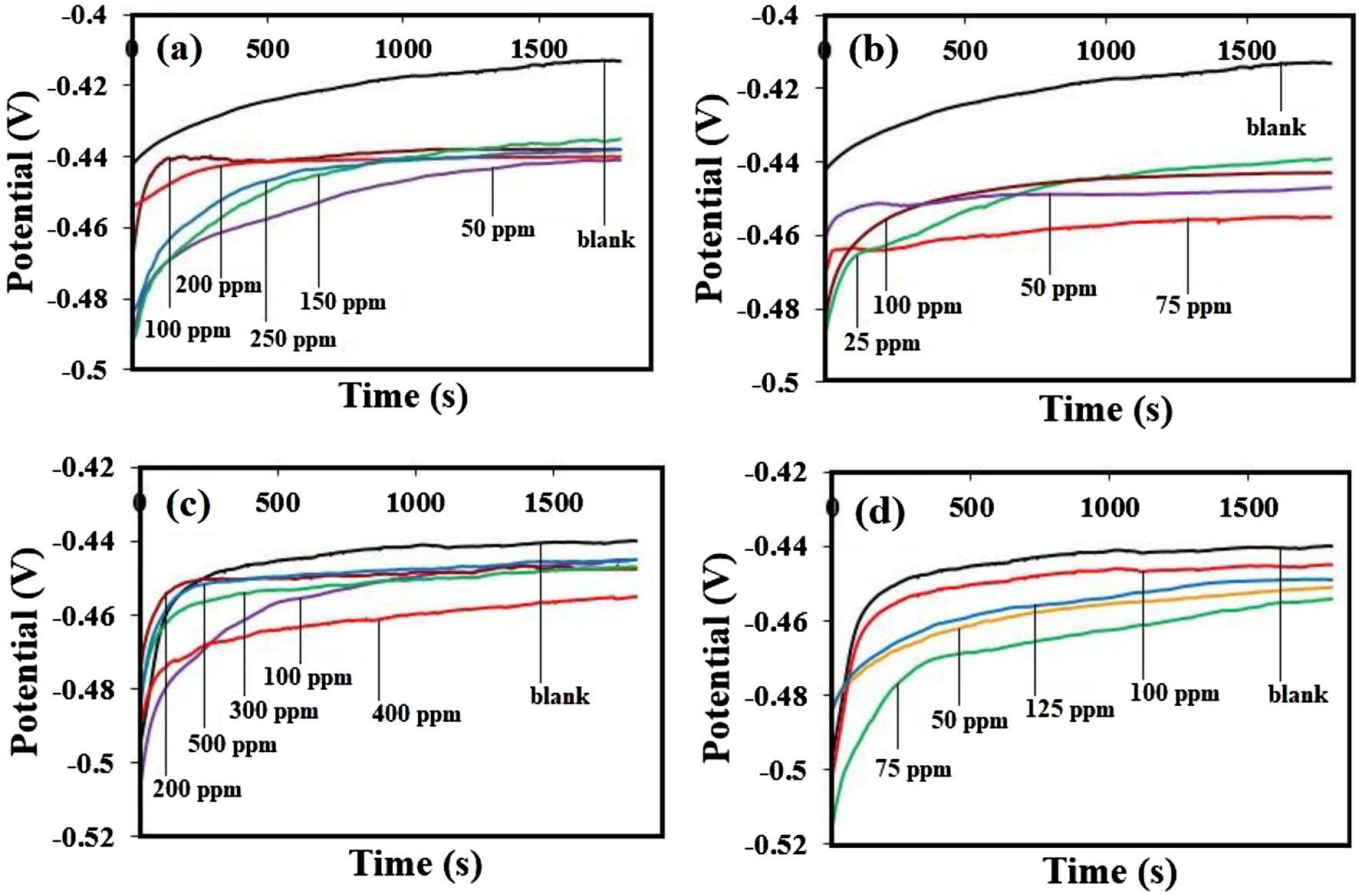
Variation of the OCP as a function of time, recorded for st-37 in 0.5 M H_2_SO_4_ (**a**) based on bulk, and (**b**) nano size of *Dracocephalum*, and in 1.0 M HCl (**c**) based on bulk, and (**d**) nano size of *Dracocephalum*, at 25 ± 1 °C.

First, by immersing the working electrode in 0.5 M H_2_SO_4_, and 1.0 M HCl solution without, and with *Dracocephalum* extract based on bulk, and nanometer size for 1800s, open-circuit potential (OCP) was stabilized (Fig. 2), and then electrochemical tests were carried out. Figure 2 illustrated that the presence of extract in acidic solutions considerably changed the OCP curves.

The corrosion behavior of st-37 in 0.5 M H_2_SO_4_, and 1.0 M HCl solutions was determined by EIS, and PP methods under different concentrations of extract based on bulk, and nano size.

#### Electrochemical impedance Spectroscopy, Bode, and Bode phase analysis in H_2_SO_4_ and HCl media

**Figure 3:**
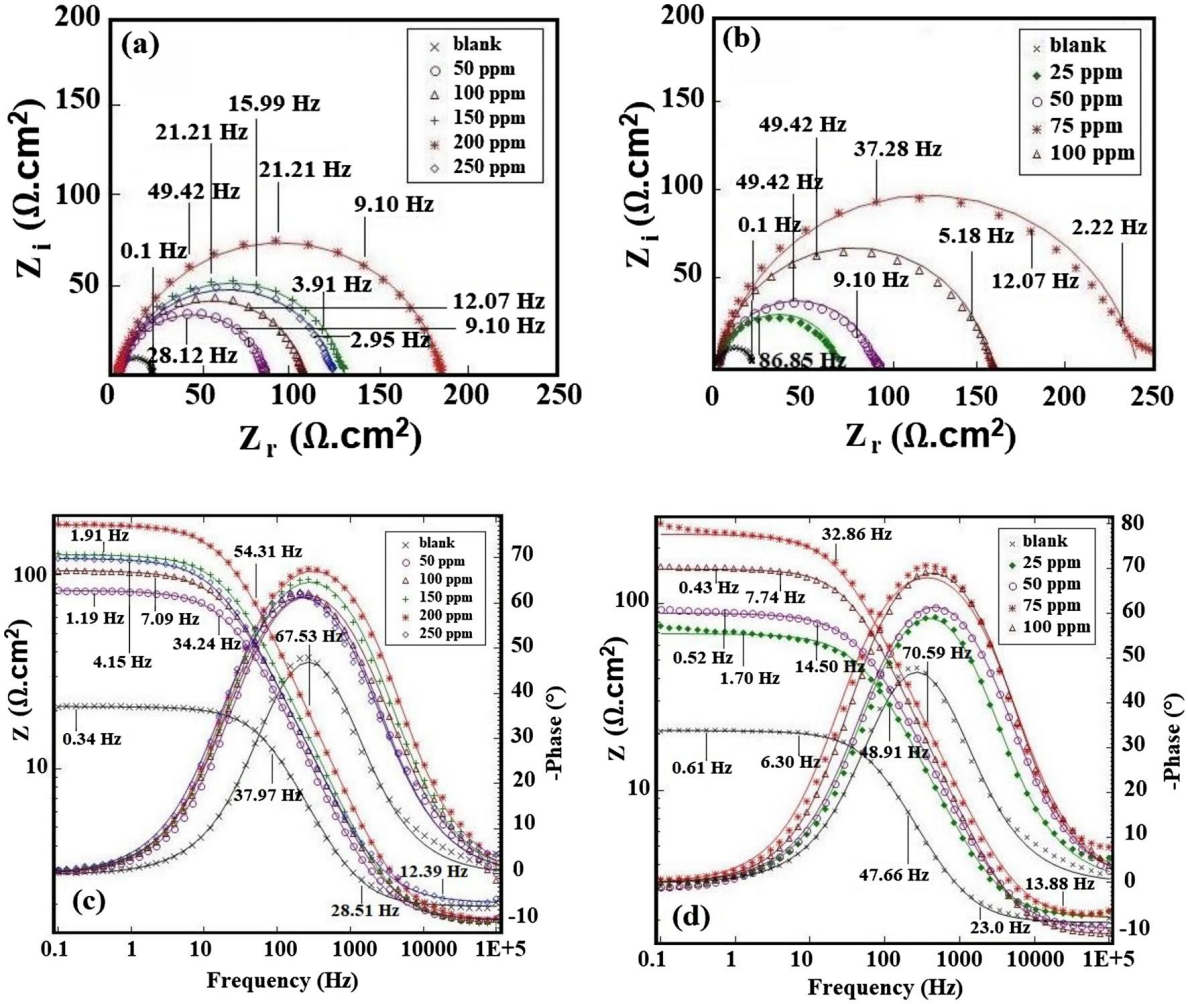
The (**a**,**b**) Nyquist plots, (**c**,**d**) bode plots, and phase angle plots for st-37 with different concentrations of *Dracocephalum* based on bulk, and nano size, in 0.5 M H_2_SO_4_.

**Figure 4:**
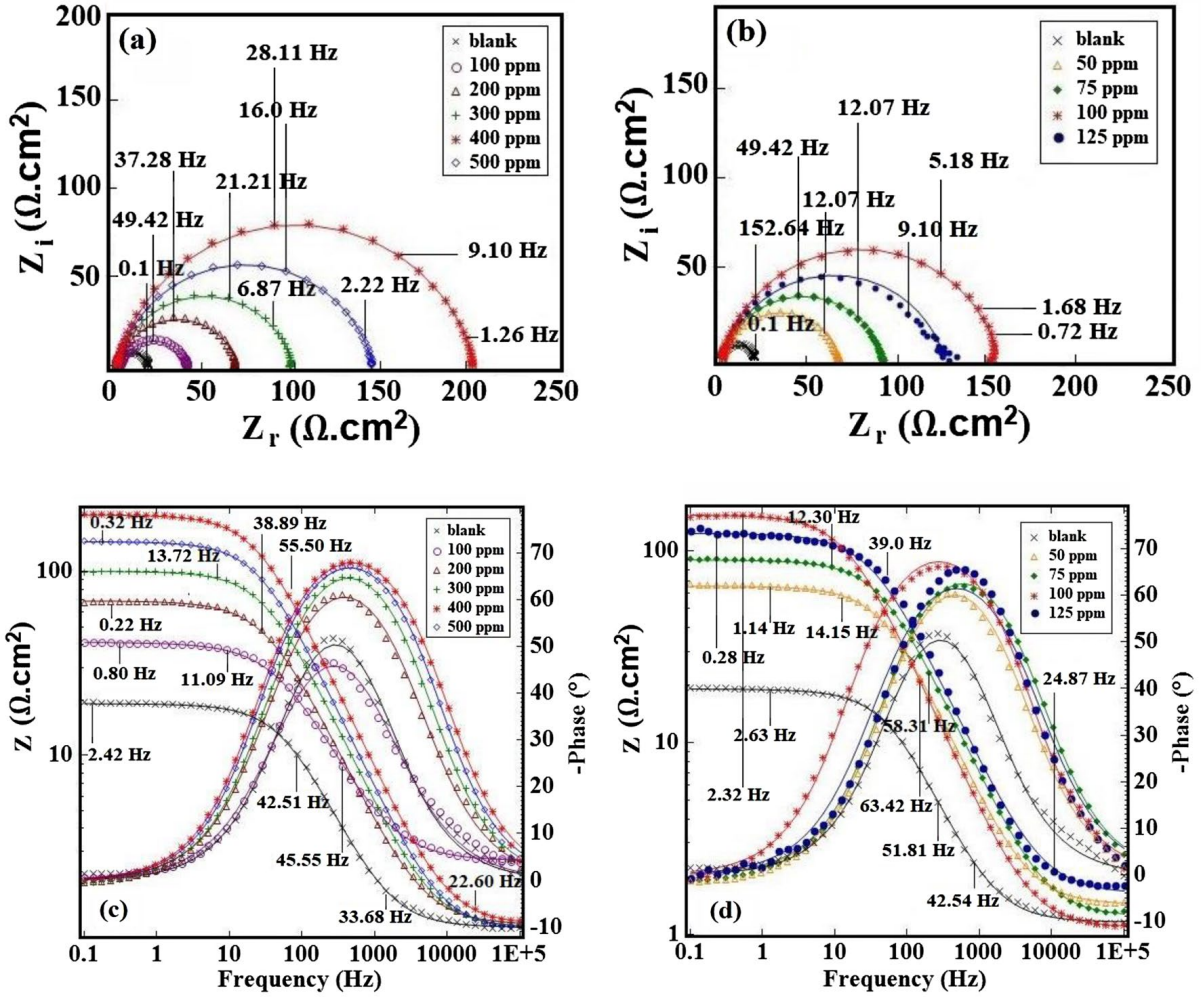
The (**a**,**b**) Nyquist plots, (**c**,**d**) bode plots, and phase angle plots for st-37 with different concentrations of *Dracocephalum* based on bulk, and nano size, in 1.0 M HCl.

The EIS is a non-destructive, and very effective method for evaluating corrosion processes at the metal-corrosive electrolyte interface. The goal of EIS is to see how different concentrations of green inhibitors affect the impedance behavior of mild steel in 0.5 M H_2_SO_4_, and 1.0 M HCl. Figures 3, and 4 demonstrate Nyquist plots, Bode plots, and changes in phase angle for st-37 in 0.5 M H_2_SO_4_, and 1.0 M HCl solutions, respectively, with varying quantities of plant extract depending on bulk, and nanosize. Only one capacitive semicircle is seen in the Nyquist plots for the examined plant extract. The existence of charge transfer resistance (*R*_*ct*_) combined with the impact of ionic double-layer capacitance (*C*_*dl*_) might explain this phenomenon^44^. The general semi-circle shape of the curves is fairly constant across the whole inhibitor concentration range, indicating that no change in corrosion mechanism has occurred as a result of the addition of plant extract^45^.

High-frequency capacitance circuits are generally generated by charge transfer resistance, as demonstrated in Figs. 3a, and 4a. It can be seen that adding inhibitor causes an increase in the radius of the capacitive ring, and inhibits electrochemical processes to some extent. It appears that adding the extract to mild steel reduces the rate of corrosion. Tables 2, and 3 show the EIS characteristics for mild steel with various concentrations of *Dracocephalum* extract (bulk, and nanosize) in acidic media, including the goodness of fit (chi-square), solution resistance (*R*_*S*_), double layer capacitance (*C*_*dl*_), charge transfer resistance (*R*_*ct*_), and the degree of surface coverage (*θ* = *IE*_*I*_/100).

**Table 2:** Corrosion parameters derived from Nyquist curves for st-37 in H_2_SO_4_ solution in the absence, and presence of different concentrations of inhibitor based on bulk, and nano size, at 25 ± 1 °C.

C/ppm	Chi-square	R_S_/Ω cm^2^	R_ct_/Ω cm^2^	C_dl_/μF cm^−2^	θ	IE%
(a) *Dracocephalum*/H_2_SO_4_ 0.5 M						
Blank	0.032	1.96	19	128	–	–
50	0.031	1.67	81	69	0.76	76
100	0.057	1.64	105	71	0.82	82
150	0.021	1.60	126	60	0.85	85
200	0.011	1.66	182	41	0.90	90
250	0.062	2.04	121	62	0.84	84
(b) *Dracocephalum* (nano)/H_2_SO_4_ 0.5 M						
25	0.065	2.08	68	47	0.72	72
50	0.024	1.80	88	37	0.78	78
75	0.16	2.06	237	32	0.92	92
100	0.019	1.69	153	28	0.88	88

**Table 3:** Corrosion parameters derived from Nyquist curves for st-37 in HCl solution in the absence, and presence of different concentrations of inhibitor based on bulk, and nano size, at 25 ± 1 °C.

C/ppm	Chi-square	R_S_/Ω cm^2^	R_ct_/Ω cm^2^	C_dl_/μF cm^-2^	θ	IE%
(a) *Dracocephalum*/HCl 1.0 M						
Blank	0.031	1.17	18	135	**–**	**–**
100	0.042	2.68	39	83	0.54	54
200	0.029	1.21	68	62	0.74	74
300	0.014	1.17	99	57	0.82	82
400	0.0082	1.21	203	37	0.91	91
500	0.012	1.13	145	40	0.88	88
(b) *Dracocephalum* (nano)/HCl 1.0 M						
50	0.029	1.44	65	50	0.72	72
75	0.014	1.24	89	36	0.80	80
100	0.036	1.08	154	65	0.88	88
125	0.24	1.75	123	35	0.85	85

These semi-circles also show that *IE*_*I*_% increases with an increase in inhibitor concentrations. It is noted that the extract with nanosize possess better *IE*_*I*_% than the bulk extract, in the same amount, in both solutions.

Also, *R*_*ct*_ increases when the concentration of *Dracocephalum* increases, owing to enhanced extract coverage on the steel surface, and higher inhibitor shielding efficiency against ion penetration of the corrosive medium^46^. When the inhibitor concentration is up to 200 ppm, and 75 ppm for 0.5 M H_2_SO_4_, and up to 400 ppm, and 100 ppm for 1.0 M HCl containing bulk, and nanosize of the extract, respectively, the *R*_*ct*_, and *IE*_*I*_% reaches the highest value (90, 92, 91, and 88%). This rise shows that the inhibitor builds an adsorption layer on the mild steel alloy's surface, preventing corrosion. *R*_*ct*_ begins to decrease as the concentration of *Dracocephalum* extract increases, as the inhibitor is desorbed from the metallic surface. As the extract concentration grew, the electric double-layer capacitor, *C*_*dl*_, dropped, which may be ascribed to a decrease in the local electric double layer constant^47^. In this case, inhibitor molecules adhered to the steel surface, and replaced the original water molecules that were present in the steel surface's interface layer. The *C*_*dl*_ decreased as the inhibitor concentration grew because the inhibitor molecules had a lower dielectric constant than water molecules, causing the inhibitor molecules to be loosely organized in the interface layer^48^. It was discovered that the extract might produce an inhibitor coating on the steel surface to prevent corrosion, indicating that *Dracocephalum* extract has high inhibition efficiency for mild steel.

The increase in phase angle with increasing extract content, as seen in the Bode plots in Figs. 3c,d, and 4c,d, further supports the prevention of corrosion^13^. The roughness of the electrode surface is linked to the value of the phase angle in these figures. The higher the value of *θ*, lower is the surface roughness. As the inhibitor concentration increases, the surface roughness decreases, implying that corrosion decreases.

**Figure 5:**
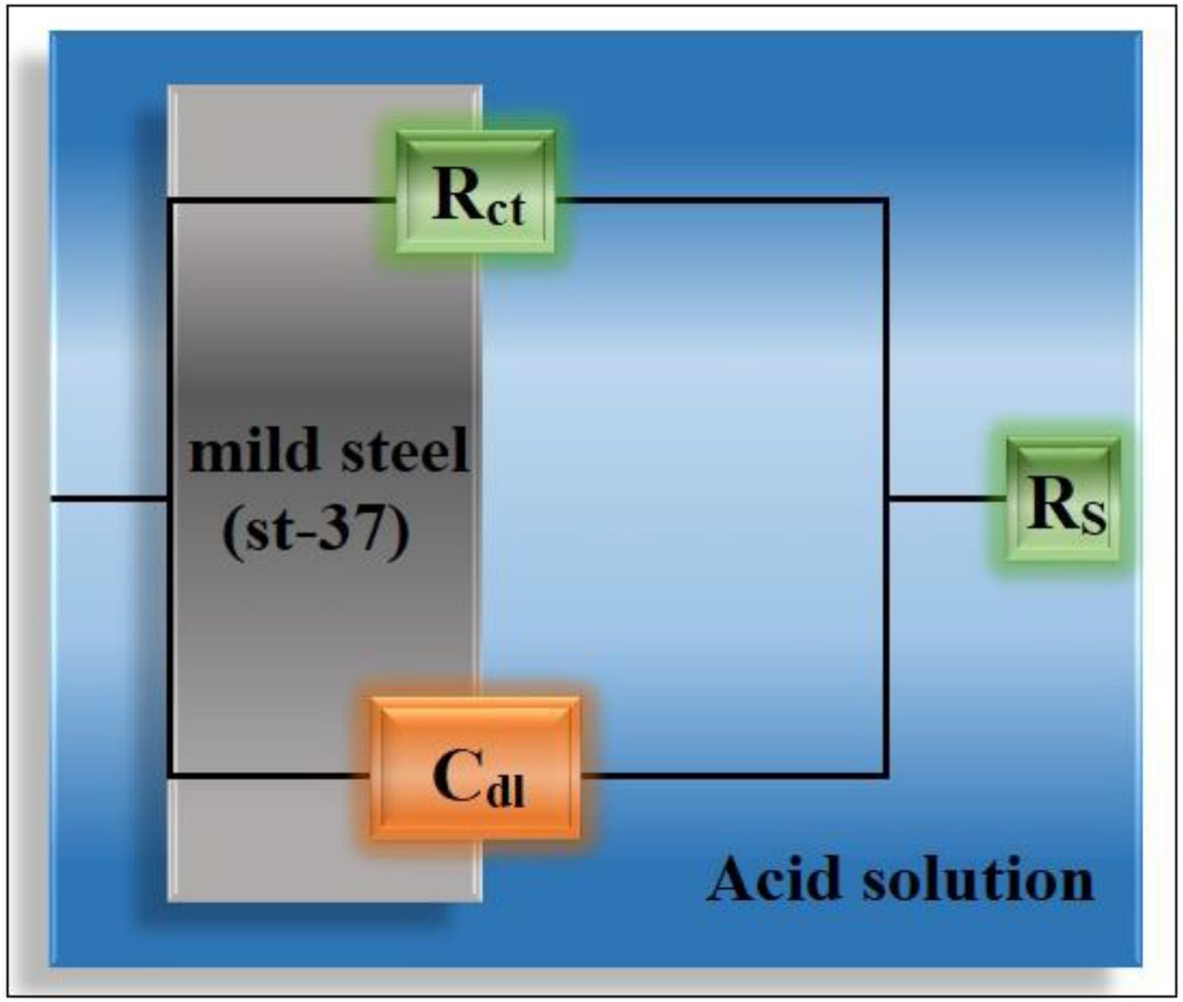
The electrical equivalent Randle's circuit model.

The equivalent Randle's circuit model (Fig. 5) was used to examine all of the impedance curves illustrated in Figs. 3, and 4. This is made up of a series solution resistance (*R*_*S*_), a parallel resistance (*R*_*ct*_), and capacitor combination (*C*_*dl*_).

#### Potentiodynamic polarization in H_2_SO_4_ and HCl media

**Figure 6:**
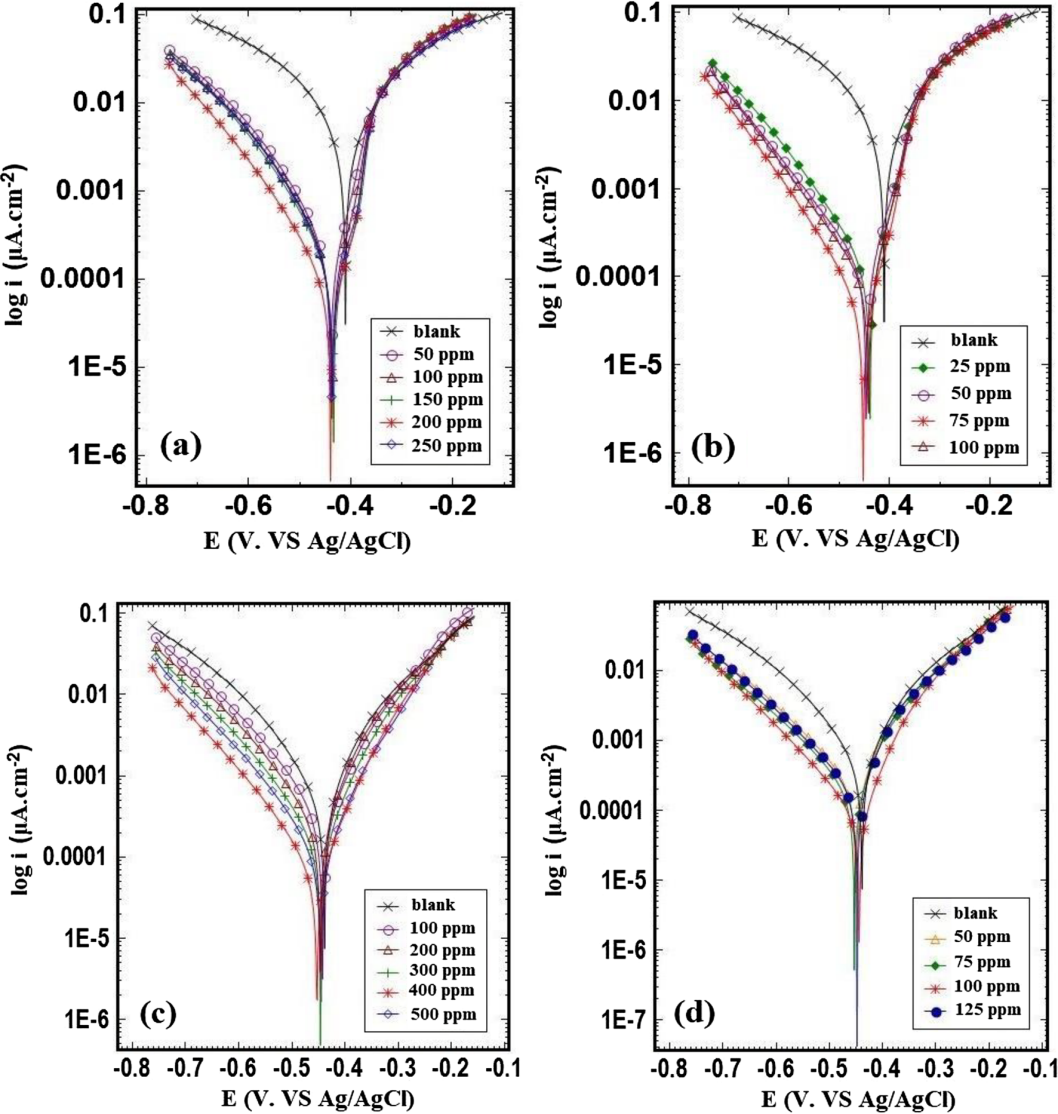
Polarization curves for st-37 in 0.5 M H_2_SO_4_ (**a**) based on bulk, and (**b**) nano size of *Dracocephalum*, and in 1.0 M HCl (**c**) based on bulk, and (**d**) nano size of *Dracocephalum*, at 25 ± 1 °C.

Figure 6 depicts the cathodic, and anodic polarization curves of mild steel following immersion in 0.5 M H_2_SO_4_, and 1.0 M HCl solutions in the absence, and presence of various amounts of extract. The experimental results including the corrosion current density (*i*_corr_), the cathodic, and anodic Tafel slopes (*β*_c_, and *β*_a_), the corrosion potential (*E*_corr_), the inhibition efficiency (*IE*_*p*_%), and the degree of surface coverage (*θ*) for different solutions are reported in Table 4. The corrosion current density was calculated using the intercept of extrapolated cathodic, and anodic Tafel lines at the corrosion potential. Also, the *IE*_*p*_% was calculated using Eq. (2).

**Table 4:** Corrosion parameters derived from polarization curves for st-37 in sulfuric acid solution with (a) inhibitor, (b) nano inhibitor, and in hydrochloric acid solution with (c) inhibitor, (d) nano inhibitor in uninhibited, and inhibited solution at 25 ± 1 °C.

C/ppm	i_corr_/μA.cm^−2^	− E_corr_/mV	β_c_/mV.dacade^−1^	β_a_/mV.dacade^-1^	**θ**	IE%
(a) *Dracocephalum*/H_2_SO_4_ 0.5 M						
Blank	1427	413	67	59	–	–
50	327	440	61	130	0.77	77
100	249	438	55	126	0.82	82
150	222	435	53	129	0.84	84
200	151	440	51	137	0.89	89
250	199	438	53	115	0.86	86
(b) *Dracocephalum* (nano)/H_2_SO_4_ 0.5 M						
25	197	439	55	143	0.86	86
50	126	447	53	136	0.91	91
75	91	454	56	135	0.94	94
100	105	443	50	135	0.93	93
(c) *Dracocephalum*/HCl 1.0 M						
Blank	752	440	106	138	–	–
100	363	445	92	127	0.52	52
200	209	446	67	111	0.72	72
300	156	447	77	117	0.79	79
400	73	455	75	117	0.90	90
500	111	445	78	119	0.85	85
(d) *Dracocephalum* (nano)/HCl 1.0 M						
50	217	451	71	126	0.71	71
75	168	454	71	125	0.78	78
100	87	445	62	120	0.88	88
125	182	449	68	128	0.76	76

From the experimental values, it can be observed that the corrosion current density decreases significantly with an increase in inhibitor concentration up to 200 ppm, and 75 ppm for 0.5 M H_2_SO_4_, and up to 400 ppm, and 100 ppm for 1.0 M HCl containing bulk, and nano size of the extract, respectively, supports the retardation of the corrosion process^49^. The reduced current density in the presence of inhibitor in all four solutions suggests that the metal surface is passivated due to the creation of the inhibitor layer^50^. The results reveal that *i*_corr_ of mild steel decreased from 1427 μA/cm to 151 μA/cm, and 1427 μA/cm to 91 μA/cm, and the *IE*% increased to 89%, and 94%, and also, 752 μA/cm to 73 μA/cm, and 752 μA/cm to 87 μA/cm, and the *IE*% increased to 90%, and 88%, for H_2_SO_4_, and HCl solutions with bulk, and nano size of the extract, respectively.

The findings of the investigation, suggest that the nano extract of the plant has greater inhibitory properties than the regular extract.

Furthermore, differences in the values of *β*_c_, and *β*_a_ compared to blank solutions show that these inhibitors safeguard the corrosion process by adsorbing inhibitor molecules on both anodic, and cathodic sites.

With the addition of inhibitors, there is a distinct change in the cathodic, and anodic parts of curves in Tafel plots of H_2_SO_4_ solution. As a result, it's referred to as a mixed-type inhibitor. From Fig. 6c,d, and Table 4, in hydrochloric acid solution, the shape of the anodic, and cathodic curves, and the Tafel parameter (*β*_*c*_, and *β*_*a*_) did not change significantly after using the extract as an inhibitor, but in sulfuric acid solution *β*_*a*_ changed (Fig. 6a,b), and this means that the inhibitor acts as a both anodic, and cathodic inhibitor (mixed one), with predominant anodic effect in H_2_SO_4_ medium. On the other hand, for H_2_SO_4_, and HCl solutions, the maximum shift in *E*_corr_ value is positive/negative side 41, and 15 mV, respectively, and a literature survey revealed that if a shift in corrosion potential is less than ± 85 mV with respect to the blank solution, the inhibitor acts as a mixed-type inhibitor; thus, this inhibitor is a mixed-type inhibitor^51^.

Based on the above analysis, the values of *IE*_*I*_%, and *IE*_*P*_% rise as the concentration of inhibitors rises, with bulk, and nano size of extract in acidic media. The mean difference between the maximum values of *%IE*_*I*_, and *%IE*_*P*_ using the best concentration of extract with bulk and nano size is 1.0, and 2.0%, in H_2_SO_4_, and 1.0, and 0.0%, in HCl solutions, respectively.

**Figure 7:**
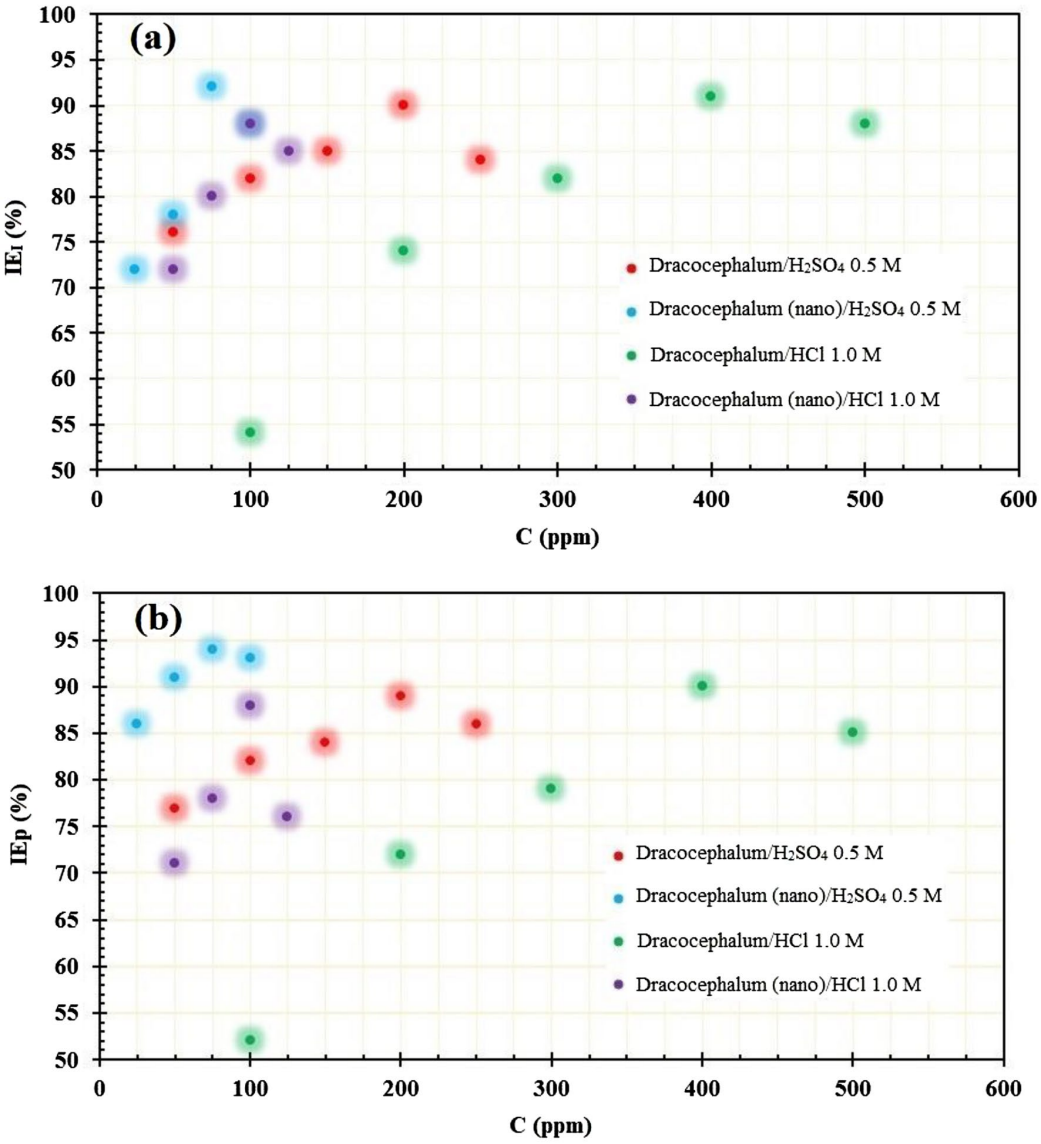
Variation of *IE*% with the concentration of inhibitor for both (**a**) impedance, and (**b**) polarization experiments.

Figure 7 shows the influence of inhibitor concentration (ppm) on inhibition efficiency (*IEp*, and *IE*_*I*_, %) for st-37 steel in 0.5 M H_2_SO_4_, and 1.0 M HCl at 25 ± 1 °C, as measured by impedance, and polarization. It has been discovered that when the concentration of extract increases, the effectiveness of inhibition increases. Inhibition efficiency significantly increases as the extract concentration increases from 0 up to 200, 75, 400, and 100 ppm in acidic media. When the concentration of inhibitor exceeds from above values, the inhibition efficiency decreases slightly. The slight change in inhibition efficiency is due to the saturation adsorption of inhibitor molecules on the alloy surface. The higher inhibition efficiency indicates that the *Dracocephalum* extract is a suitable corrosion inhibitor for both acidic media.

### Adsorption isotherm

Adsorption isotherms serve a critical function in providing extensive information about the current interaction behavior between metal surfaces, and *Dracocephalum* extract molecules^52^.

**Figure 8:**
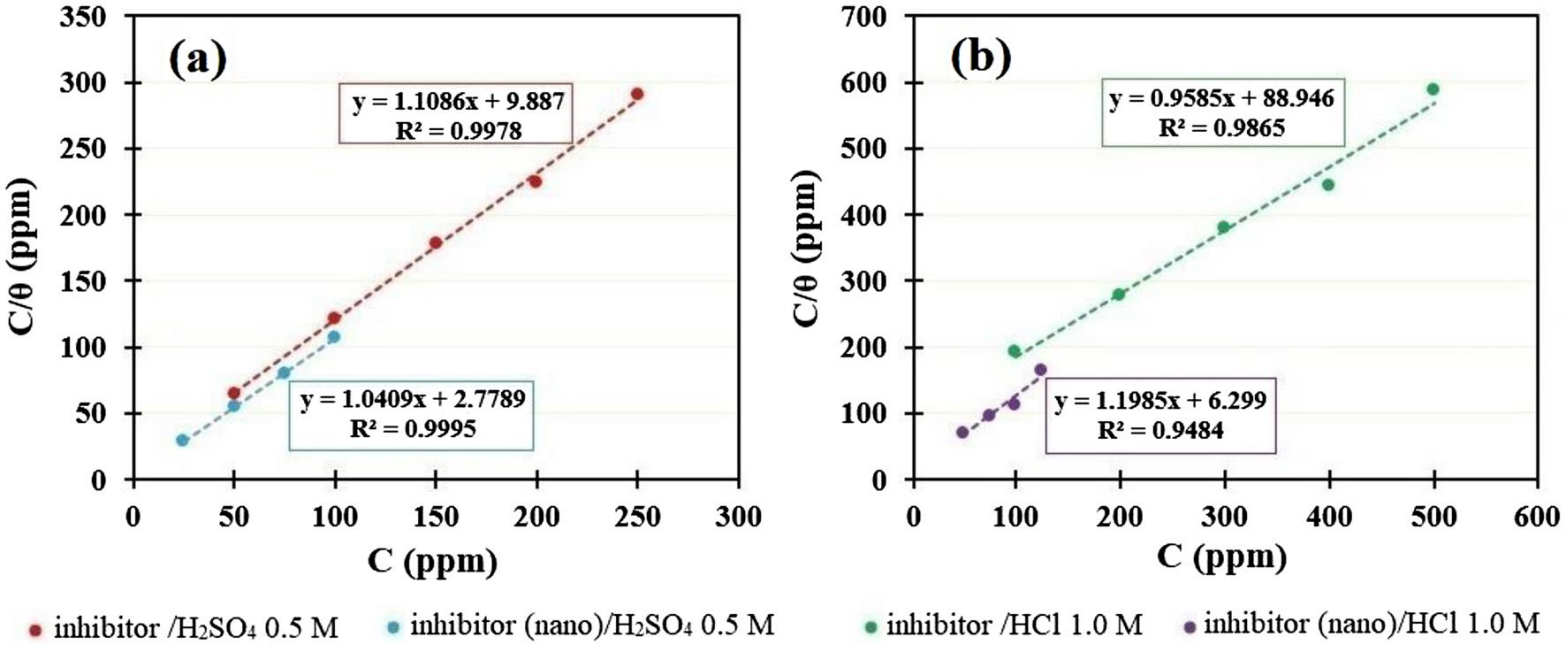
Langmuir adsorption isotherm of the inhibitor determined by Tafel polarization data for st-37 in (**a**) 0.5 M H_2_SO_4_, and (**b**) 1.0 M HCl solutions at 25 ± 1 °C.

Different adsorption isotherm models were utilized in this work to suit the experimental results. The Langmuir isotherm is in good agreement with the experimental findings. The general form of the Langmuir isotherm model is shown in below equation^53–57^:

3 $$\frac{C}{\theta }=\frac{1}{{K}_{ads}}+C$$

where, $\theta$, *K*_*ads*_, and *C* are the metal surface coverage, the equilibrium constant for the adsorption–desorption process, and the inhibitor concentration, respectively. As can be seen, when a graph is drawn between (*C/θ*), and *C*, a straight line (*R*^2^ > 0.9) is formed for all samples, as shown in Fig. 8, with a gradient (slope) near to the unit and an intercept equal to *K*_*ads*_. The fact that all linear correlation coefficients (*R*) are almost equal to one shows that plant extract adsorption on mild steel surfaces follows the Langmuir adsorption isotherm. The Langmuir isotherm implies inhibitor molecule monolayer adsorption, or the inhibitor molecule occupies one active site on a metal surface^58^. Furthermore, the Langmuir adsorption isotherm revealed that organic components in plant extracts with polar atoms or groups adsorbed on the metal surface may interact via mutual attraction or repulsion^59^.

The calculated adsorption coefficient, *K*_*ads*_, was larger in H_2_SO_4_ than in HCl, indicating that the adsorption of inhibitor molecules on active sites of steel surfaces was easier in H_2_SO_4_ than in HCl solution^60^. The strength, and stability of the adsorbed layer formed by nano extract in both solutions could also be evaluated from the higher *K*_*ads*_ value compared to the other situation.

The standard adsorption free energy (${\Delta G}_{ ads}^{o}$) is also calculated using the *K*_*ads*_ values. In the context of corrosion inhibition, physisorption, and chemisorption are two adsorption mechanisms that are frequently studied^61^. For the physical adsorption, values of the standard adsorption free energy are until − 20 kJ/mol, while those lower than − 40 kJ/mol are correlated with the chemical adsorption^3,53^.

${\Delta G}_{ ads}^{o}$ of the adsorption process linked with *K*_*ads*_, and determined using below equation^62^:

4 $${{K}_{ads}}=1/ 10^6 exp (\frac{-{\Delta G}_{ ads}^{o}}{RT})$$

where, *R*, and *T* are the universal gas constant, and thermodynamic temperature, respectively, and 10^6^ points to the ppm concentration (mg/L) of water.

For H_2_SO_4_ solution with bulk, and nano size of extract the calculated value of ${\Delta G}_{ ads}^{o}$ is 28.54, and − 31.71 kJ/mol, respectively. For HCl solution with bulk, and nano size of extract, the calculated value of ${\Delta G}_{ ads}^{o}$ is 22.83, and − 29.70 kJ/mol, respectively. As a result of the obtained value for ${\Delta G}_{ ads}^{o}$, it can be concluded that *Dracocephalum* adsorption is not solely chemisorption or physisorption, but also includes comprehensive adsorption (both chemical and physical), and that the negative sign of ${\Delta G}_{ ads}^{o}$ indicates that inhibitor molecule adsorption on the metal surface is spontaneous^3^. Table 5 lists the results, including *K*_*ads*_, and ${\Delta G}_{ ads}^{o}$.

**Table 5:** Thermodynamic characteristics of adsorption obtained by PP measurements for st-37 in acidic media in the presence of varying concentration of extract.

Sample	K_ads_ (L/mg)	${\Delta \mathrm{G}}_{\mathrm{ ads}}^{\mathrm{o}}$(kJ/mol)
*Dracocephalum*/H_2_SO_4_	0.10	− 28.54
*Dracocephalum* (nano)/H_2_SO_4_	0.36	− 31.71
*Dracocephalum* /HCl	0.01	− 22.83
*Dracocephalum* (nano)/HCl	0.16	− 29.70

A comparison of the present research with similar studies that have used plant extract as a corrosion inhibitor in acidic media presented in Table 6. It can be concluded that *Dracocephalum* extract in bulk, and especially in nanometer size is a suitable candidate for boosting the corrosion resistance of mild steel alloy in 0.5 M H_2_SO_4_, and 1.0 M HCl.

**Table 6:** Summary of similar studies for plant extract as a corrosion inhibitor and in comparison to this study.

Refs	inhibitor	Concentration of inhibitor	alloy	Corrosive medium	PCE (%)
^63^	*Origanum Compactum* extract	400 ppm	mild steel	1.0 M HCl	90
^64^	*Ammi visnaga* extract	700 ppm	Carbon steel	1.0 M HCl	84
^65^	*Quince seed* extract	800 ppm	Mild steel	1.0 M HCl	95
^66^	*Inula viscosa* leaves extract	600 ppm	Carbon steel	1.0 M HCl	92
^67^	*Mish Gush* leaves extract	1200 ppm	Mild steel	1.0 M HCl	96
^68^	*Sugarcane purple rind* extract	800 ppm	Carbon steel	1.0 M HCl	96.2
^69^	*Artabotrys odoratissimus* extract	1250 ppm	Mild steel	0.5 M H_2_SO_4_	93
^70^	*Cinnamoum tamala* extract	100 ppm	Carbon steel	0.5 M H_2_SO_4_	96.76
This work	*Dracocephalum* extract	200 ppm	Mild steel	0.5 M H_2_SO_4_	89
This work	*Dracocephalum* extract (nano)	75 ppm	Mild steel	0.5 M H_2_SO_4_	94
This work	*Dracocephalum* extract	400 ppm	Mild steel	1.0 M HCl	90
This work	*Dracocephalum* extract (nano)	100 ppm	Mild steel	1.0 M HCl	88

### Mechanism of corrosion inhibition

**Figure 9:**
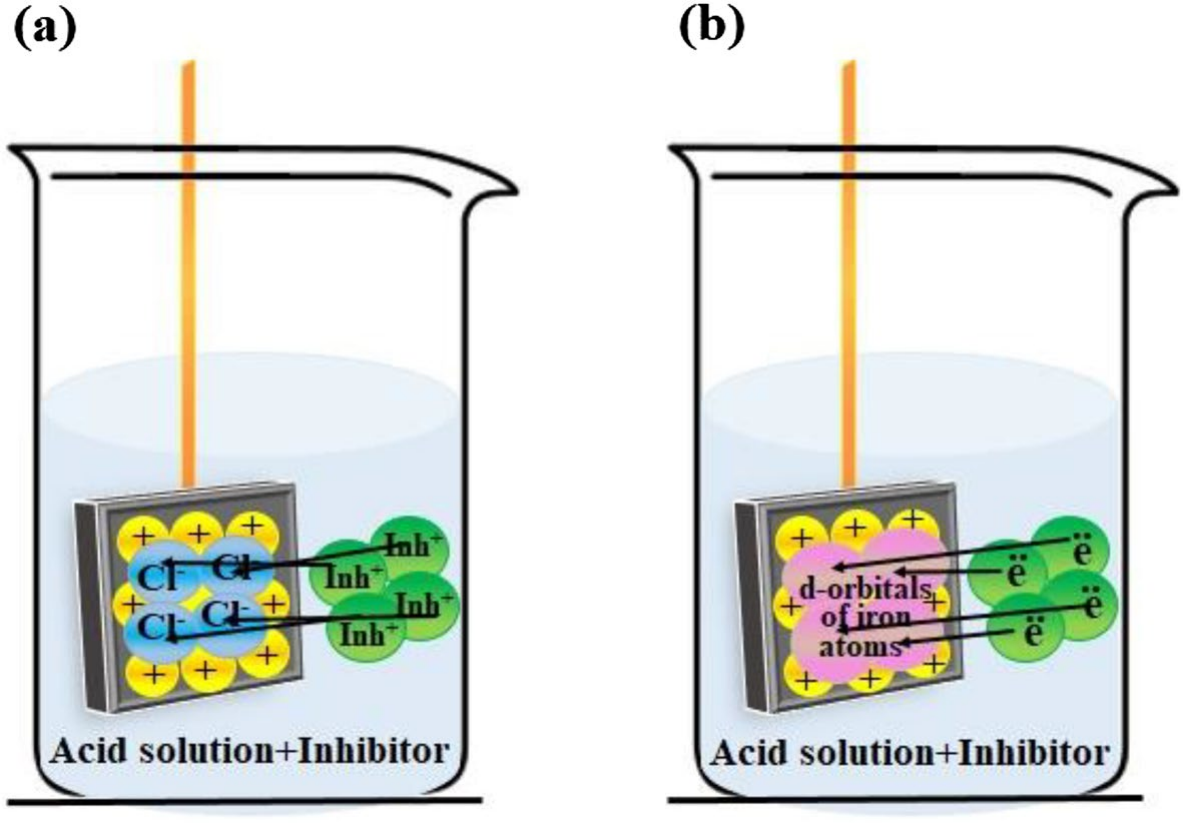
Mechanism of corrosion inhibition adsorption on the metal surface: (**a**) physical adsorption, and (**b**) chemical adsorption.

The examined compounds' ability to prevent carbon steel corrosion is mostly owing to their physical or chemical adsorption on the metal surface, where they replace H_2_O molecules on the steel surface, and form a compact barrier coating^71^. Electrostatic contact occurs between charged inhibitor molecules, and charged metal surfaces in the event of physical adsorption (Fig. 9a). During chemical adsorption, the pair electron on the π-electron of multiple bonds, and heteroatoms interact with the iron's unoccupied d-orbitals (Fig. 9b)^13^. In this work, the values of ${\Delta G}_{ ads}^{o}$ are − 22.83, and 29.70 kJ mol^−1^, in HCl solution, indicating that the examined compound molecules are adsorbed by a mix of chemical, and physical adsorption. It is known experimentally that the steel surface is positively charged in acidic solutions, Cl^−^ ions may be adsorbed on the positively charged steel surface, and subsequently, the protonated inhibitor molecules are adsorbed via electrostatic attraction (physical adsorption). But at the same time, d-orbitals of iron atoms get a lone pair of electrons on π-electron, and heteroatoms in the extract structure (Chemical adsorption). In the H_2_SO_4_ solution, the values of ${\Delta G}_{ ads}^{o}$ are -28.54, and 31.71 kJ mol^−1^, but due to the low electron charge density on the surface of ${\mathrm{SO}}_{4}^{2-}$ ions, the examined compound molecules are more adsorbed by chemical adsorption.

### UV–visible reflection measurements

**Figure 10:**
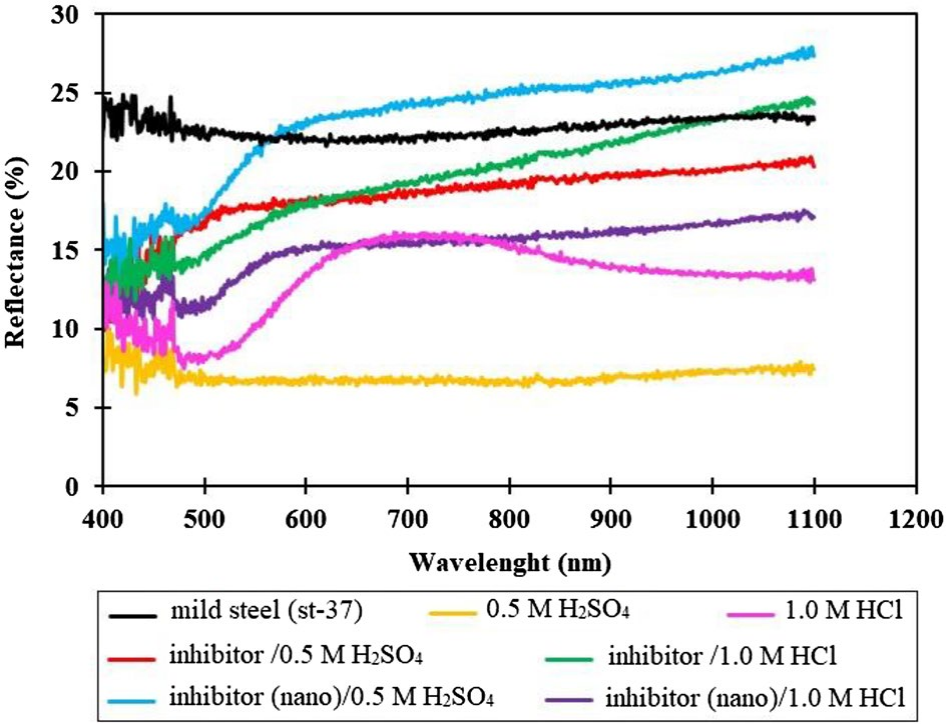
UV-Vis spectrum for st-37 specimens in 0.5 M H_2_SO_4_, and 1.0 M HCl solutions in the absence, and presence of an optimum concentration of *Dracocephalum* extract as a corrosion inhibitor.

The present surface analysis gives the reflectance of metal specimens before, and after immersion in 0.5 M H_2_SO_4_, and 1.0 M HCl in the absence, and the presence of the best inhibitor concentration. The results shown in Fig. 10 indicate that the reflectance of the st-37 specimen has decreased after immersion in acidic media in the absence of extract. Whereas, adding the corrosion inhibitor to the test solution increases the reflectance value until it is close to the specimen reflectance before immersion in the acidic solution.

### Scanning electron and optical microscopic observations

**Figure 11:**
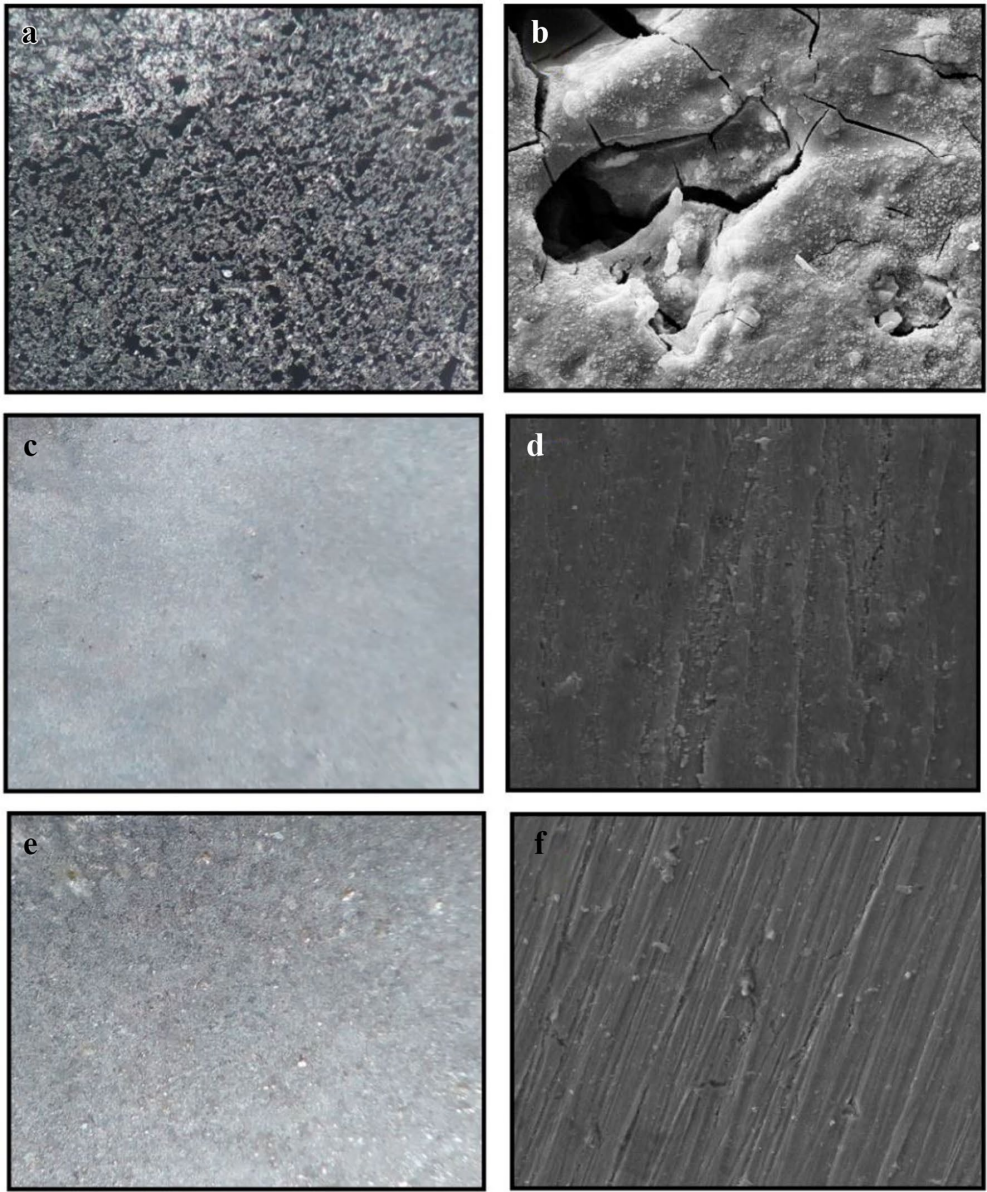
The images of the st-37 surface after 24 h immersion in 0.5 M H_2_SO_4_ solution in the (**a**,**b**) absence, (**c**,**d**) presence of 200 ppm of *Dracocephalum* extract in bulk size (**e**,**f**), and presence of 75 ppm of *Dracocephalum* extract in nano size, using optical, and Scanning electron microscopy, respectively.

**Figure 12:**
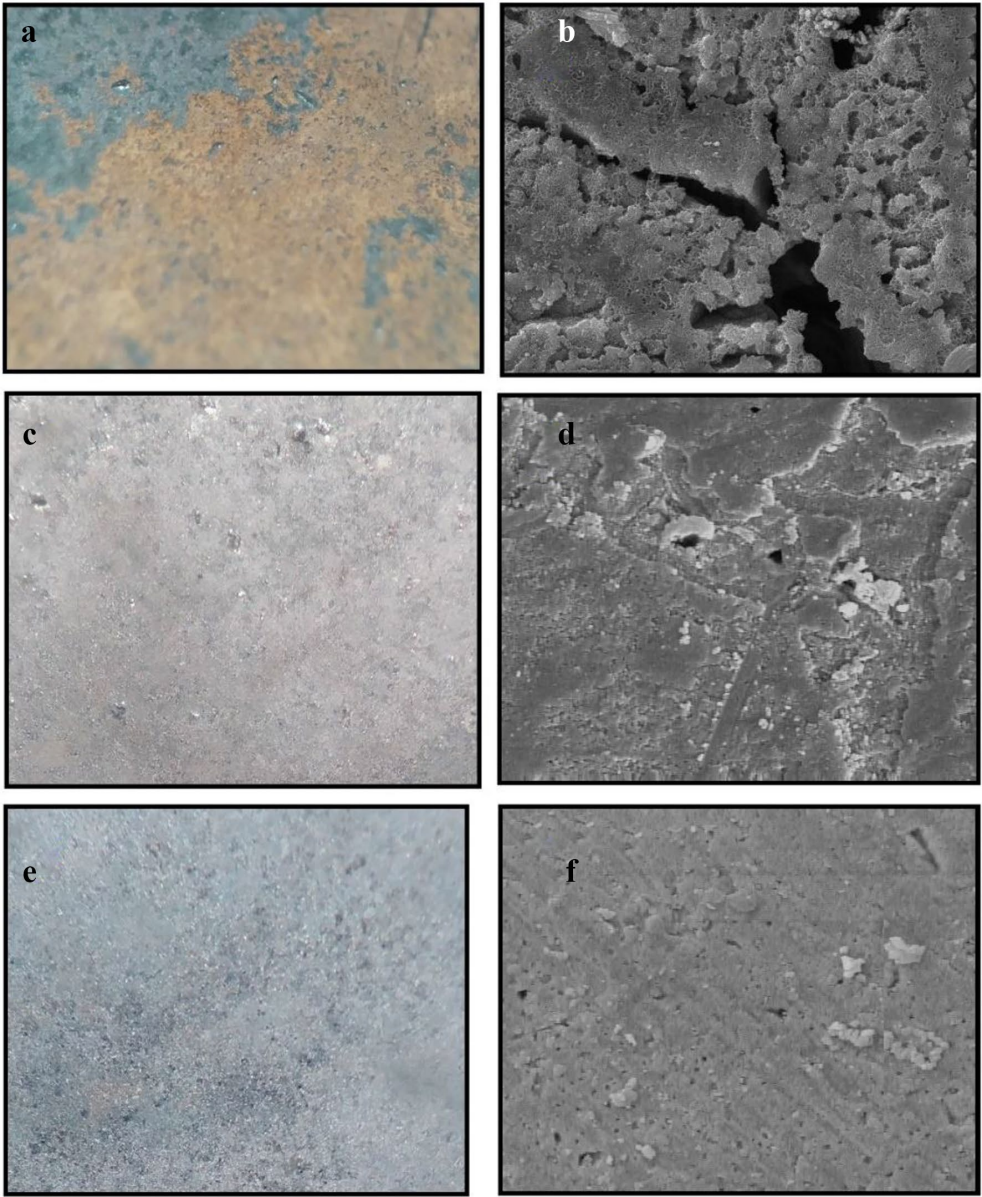
The images of the st-37 surface after 24 h immersion in 1.0 M HCl solution in the (**a**,**b**) absence, (**c**,**d**) presence of 400 ppm of *Dracocephalum* extract in bulk size (**e**,**f**), and presence of 100 ppm of *Dracocephalum* extract in nano size, using optical, and Scanning electron microscopy, respectively.

In Figs. 11, 12, optical microscopy, and scanning electron micrographs, were used to understand the surface morphology of mild steel after 24 h of immersion in 0.5 M H_2_SO_4_, and 1.0 M HCl, without, and with the best concentration of extract. In the case of blank solutions, Figs. 11(a,b) and 12(a,b) reveal a highly rough the specimen surface with serious damage, obvious pits, and cracks. However, after adding a corrosion inhibitor to the acidic media, the corrosion was visibly reduced, and the surface of the samples became reasonably smooth (Figs. 11(c–f) and 12(c–f), the corrosion inhibition efficacy showed up, and the protective inhibitor coating was produced. Figures 11(e,f) and 12(e,f) illustrate that in the presence of the optimal inhibitor concentration depending on nano size, surface corrosion of the alloy decreased substantially. It confirms that the *Dracocephalum* extract (nano) molecules cover the metal surface better than the bulk size. When comparing the images related to both solutions, it can be observed that the extract as a corrosion inhibitor in H_2_SO_4_ has a better impact than the HCl solution.

## Conclusions

The effect of *Dracocephalum* extract based on bulk, and nanometer size as a corrosion inhibitor for mild steel in 0.5 M H_2_SO_4_, and 1.0 M HCl solutions was investigated:

1. The data derived from EIS, and PP curves indicate that the inhibition efficiency augmented with the increase in extract concentration up to a special dose.
2. By polarization method, in HCl solution, the highest *IE*% is 88% at the best dose of nano extract (100 ppm), but the highest *IE*% is 90% at best dose of the bulk extract (400 ppm). In the H_2_SO_4_ solution, the highest *IE*% is 89% at the best dose of the bulk extract (200 ppm), but the corrosion inhibitor had the best inhibition efficiency (94%), at the minimum concentration (75 ppm) of nano extract. It was worth noting that the value of *IE*% calculated by PP shows the same trend as that obtained from the EIS curves method.
3. In both acidic environments, PP measurements reveal that this examined chemical reduced corrosion by mixed-type inhibition, impacting both hydrogen evolution, and metal dissolution, with a predominant anodic action in the H_2_SO_4_ medium.
4. According to EIS, this compound reduced corrosion through adsorption on the metal/solution contact.
5. ${\Delta G}_{ ads}^{o}$ suggested that *Dracocephalum* adsorption is not only chemisorption or physisorption but also includes comprehensive adsorption. That means, the investigated compound adsorbed both chemical, and physical adsorption on the st-37 surface while following the Langmuir isotherm. Furthermore, the negative value of ${\Delta \mathrm{G}}_{\mathrm{ ads}}^{\mathrm{o}}$ shows that inhibitor molecules adsorb spontaneously on the metal surface.
6. Optical, and SEM microscopy were used to confirm the corrosion testing. Thus, a uniform and less damaged surface was found with the optimal concentration of *Dracocephalum* extract in both acid solutions. The corrosion inhibition effectiveness showed up, and the protective inhibitor film was formed.

Finally, compared to the results of other researchers, it can be concluded that the *Dracocephalum* extract has the lowest optimal concentration, and proper efficiency. Therefore, by using *Dracocephalum* extract based on nanometer size, we could reduce the optimal concentration of inhibitor significantly, and increase the corrosion resistant, as well as efficiency. That is a cheap, eco-friendly, and efficient method to reduce the corrosion of mild steel in acidic media. So, *Dracocephalum* extract can be a suitable candidate for boosting the corrosion resistance of mild steel alloy in 0.5 M H_2_SO_4_, and 1.0 M HCl.

## Data Availability

The datasets used and/or analyzed during the current study available from the corresponding author on reasonable request.
